# Effect of vaccine dose on the safety and immunogenicity of a candidate TB vaccine, MVA85A, in BCG vaccinated UK adults

**DOI:** 10.1016/j.vaccine.2012.06.084

**Published:** 2012-08-17

**Authors:** Ansar A. Pathan, Angela M. Minassian, Clare R. Sander, Rosalind Rowland, David W. Porter, Ian D. Poulton, Adrian V.S. Hill, Helen A. Fletcher, Helen McShane

**Affiliations:** The Jenner Institute, University of Oxford, Oxford, United Kingdom

**Keywords:** Tuberculosis, Vaccine, BCG, MVA

## Abstract

**Purpose:**

A non-randomised, open-label, Phase I safety and immunogenicity dose-finding study to assess the safety and immunogenicity of the candidate TB vaccine Modified Vaccinia virus Ankara expressing Antigen 85A (MVA85A) from *Mycobacterium tuberculosis* (MTB) in healthy adult volunteers previously vaccinated with BCG.

**Methods:**

Healthy BCG-vaccinated volunteers were vaccinated with either 1 × 10^7^ or 1 × 10^8^ PFU of MVA85A. All adverse events were documented and antigen specific T cell responses were measured using an ex vivo IFN-γ ELISPOT assay. Safety and immunogenicity were compared between the 2 dose groups and with a previous trial in which a dose of 5 × 10^7^ PFU MVA85A had been administered.

**Results:**

There were no serious adverse events recorded following administration of either 1 × 10^7^ or 1 × 10^8^ PFU of MVA85A. Systemic adverse events were more frequently reported following administration of 1 × 10^8^ PFU of MVA85A when compared to either 5 × 10^7^ or 1 × 10^7^ PFU of MVA85A but were mild or moderate in severity and resolved completely within 7 days of immunisation. Antigen specific T cell responses as measured by the IFN-γ ELISPOT were significantly higher following immunisation in adults receiving 1 × 10^8^ PFU compared to the 5 × 10^7^ and 1 × 10^7^ doses. Additionally, a broader range of Ag85A epitopes are detected following 1 × 10^8^ PFU of MVA85A.

**Conclusion:**

A higher dose of 1 × 10^8^ PFU of MVA85A is well-tolerated, increases the frequency of IFN-γ secreting T cells detected following immunisation and broadens the range of Ag85A epitopes detected.

## Highlights

► We compared 3 doses of a the candidate TB vaccine MVA85A. ► All doses of the vaccine were safe and induced a Th1 type immune response. ► The strongest and most sustained response was seen with the highest dose of MVA85A. ► A high dose of 1 × 10^8^ PFU of MVA85A is safe and induces sustained immunity.

## 1 Introduction

TB remains one of the world's most serious infectious diseases and is responsible for more than 2 million deaths each year [1]. The only available vaccine, *Mycobacterium bovis* Bacille Calmette Guérin (BCG), confers some protection against disseminated TB in childhood but is largely ineffective at protecting against adult pulmonary disease [2]. Thus, a more effective TB vaccine is urgently needed. New vaccines for TB are assessed on measures including safety, the ability to confer protection against *Mycobacterium tuberculosis* (*MTB*) challenge in preclinical animal models, and the ability to induce an antigen specific IFN-γ immune response. Although there is no immune correlate of protection for TB, impairment of IFN-γ and IL-12 signalling in humans is associated with susceptibility to mycobacterial disease and the measurement of antigen specific IFN-γ remains the primary immune outcome in Phase I testing of new TB vaccine candidates [3].

We have previously reported that recombinant Modified Vaccinia virus Ankara (MVA) expressing antigen 85A from *MTB* (MVA85A) is well-tolerated and enhances the frequency of antigen-specific IFN-γ producing T cells in adults, children and infants previously vaccinated with BCG [4–10]. We have also shown that antigen specific T cells induced by MVA85A are highly polyfunctional, and can express IFN-γ, TNF-α, IL-2, MIP1-β and IL-17 [11,12]. However, to date we have not performed any dose-finding studies in UK adults. We therefore conducted a dose finding study in UK adults to further understand the relationship between dose and immunogenicity.

In South African infants the magnitude of the immune response to MVA85A was lower than previously reported for adults from the same population and was not increased by administration of a higher dose [4]. In the present study we have compared the magnitude and breadth of the T cell response induced by 1 × 10^7^, 5 × 10^7^ and 1 × 10^8^ plaque forming units (PFU) of MVA85A and have shown that both are greater at 12 months following immunisation in adults receiving a high dose of 1 × 10^8^ PFU of MVA85A.

## 2 Methods

### 2.1 Ethics statement

Participants were recruited under a protocol approved by the Oxfordshire Research Ethics Committee (OxREC A), ClinicalTrials.gov ID NCT00465465. Written informed consent was obtained from all individuals prior to enrolment in the trial.

### 2.2 Study design and participants

**Fig. 1:**
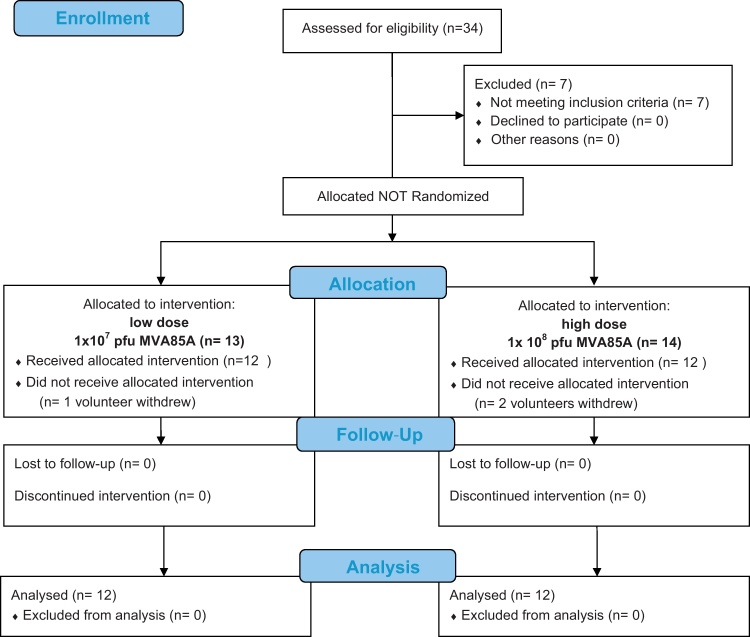


This was a non-randomised, open-label, Phase I safety and immunogenicity dose-finding study in healthy, previously BCG-vaccinated adults (Fig. 1). Participants were negative for HIV, HBV and HCV and aged 18–50 with no evidence of latent *MTB* infection, as determined by IFN-γ ELISPOT response to ESAT-6 and CFP-10.

Volunteers were vaccinated with a single immunisation of MVA85A, administered intradermally over the deltoid region of the arm. The first 12 participants enrolled received the higher dose, 1 × 10^8^ PFU of MVA85A and the following 12 participants received 1 × 10^7^ PFU of MVA85A.

### 2.3 Follow up and safety measures

Safety was assessed by monitoring blood parameters using routine haematology and biochemistry assays at weeks 1 and 12 following immunisation. In addition, a diary card was completed by all volunteers recording temperature and local and systemic adverse events for 7 days following immunisation. Participants returned for safety and immunological follow-up at 2 days, and 1, 2, 4, 8, 12, 24 and 52 weeks following immunisation. Adverse events (AE) were graded as absent, mild, moderate or severe. A moderate AE was defined as having some impact on daily activity with no or minimal medical intervention or therapy required whereas a severe AE was defined as an AE which restricted daily activity, with medical intervention or therapy required.

### 2.4 Immunological assays

As with previous trials of MVA85A, the primary assay used to measure immunogenicity was the ex vivo IFN-γ ELISPOT assay used as previously described [9]. Antigen specific responses were assessed by culturing PBMC (0.3 × 10^6^) overnight for 18 h with 20 μg/ml purified protein derivative (PPD), 10 μg/ml recombinant Ag85A protein or pools of Ag85A peptides (10 μg/ml each peptide) overlapping by 10 amino acids (Table 1). Blood samples for IFN-γ ELISPOT were collected on the day of immunisation and 1, 2, 4, 8, 12, 24 and 52 weeks following immunisation. A

### 2.5 Data analysis

Data was analysed using Stata software (StataCorp). As immune data was available from multiple time-points, an area under the curve (AUC) analysis was performed to obtain a value for overall immune response to MVA85A. Kruskal–Wallis and Mann–Whitney tests were used to determine significant differences in immunogenicity between doses. Wilcoxon signed rank was used to determine differences within a dosing group. Data was compared between the 2 doses evaluated in this trial and with a previously published trial where a dose of 5 × 10^7^ PFU of MVA85A had been administered [9].

## 3 Results

### 3.1 Participants

There were 24 participants enrolled into the study, 12 received 1 × 10^7^ PFU MVA85A and 12 received 1 × 10^8^ PFU of MVA85A. Demographic characteristics are summarised in Table 2. There were an equal number of males (33%) in each dosing group which was equivalent to previous trials with MVA85A [9] (Table 2). A higher proportion of participants were either healthcare workers or born outside of the UK when compared to previous studies with MVA85A.

### 3.2 Safety of MVA85A

**Fig. 2:**
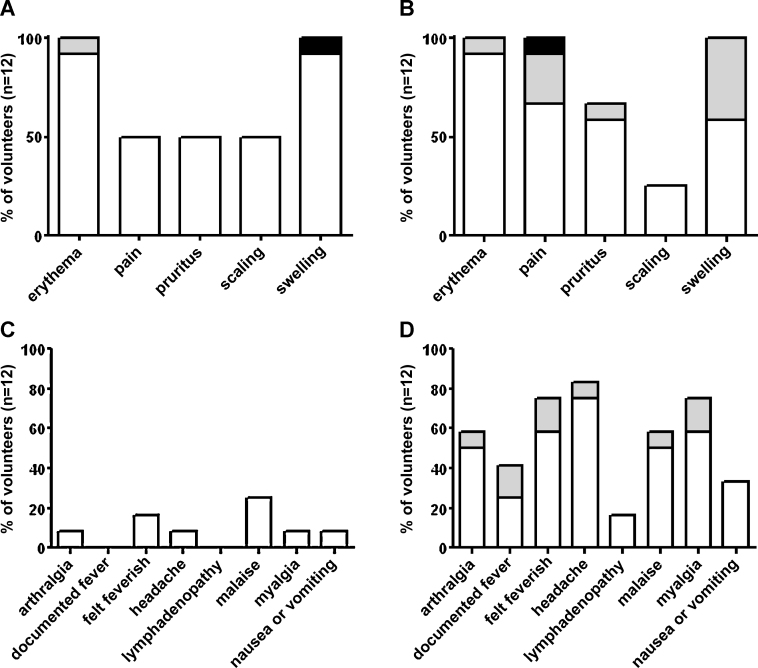
Severity of adverse events induced by immunisation with different doses of MVA85A. Severity of the most frequently reported local adverse events following immunisation with (A) 1 × 10^7^ or (B) 1 × 10^8^ PFU of MVA85A. Severity of the most frequently reported systemic adverse events following immunisation with (C) 1 × 10^7^ or (D) 1 × 10^8^ PFU of MVA85A. White: mild, grey: moderate and black: severe.

The profiles of reported local AEs were similar across the two doses tested, except for a lower frequency of pain recorded for the 1 × 10^7^ PFU group (Table 3). Local AEs were either mild or moderate with the exception of one report of severe swelling in the 1 × 10^7^ PFU group and one report of severe pain in the 1 × 10^8^ PFU group (Fig. 2A and B). The local AE profile was comparable to that previously reported for a dose of 5 × 10^7^ PFU of MVA85A [9] (Table 3). Systemic AEs were more frequently reported by participants receiving the 1 × 10^8^ PFU dose of MVA85A when compared to the 1 × 10^7^ and 5 × 10^7^ PFU groups. However all systemic AEs were recorded as either mild or moderate in severity (Fig. 2C and D).

### 3.3 Effect of MVA85A dose on magnitude and longevity of T cell response

**Fig. 3:**
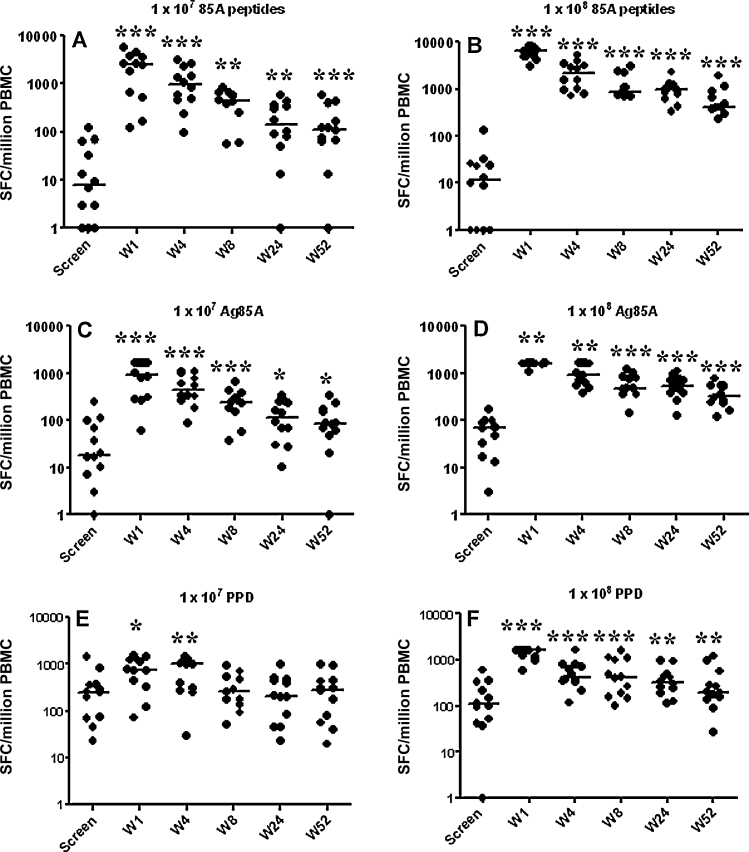
T cell responses in adults vaccinated with either 1 × 10^7^ or 1 × 10^8^ PFU of MVA85A. Antigen specific T cells were detected in PBMC from healthy, previously BCG vaccinated adults receiving either 1 × 10^7^ (panels A, C and E) or 1 × 10^8^ (panels B, D and F) PFU of MVA85A. T cells were detected using an overnight ex vivo IFN-γ ELISPOT assay with summed peptide pool (A and B), Ag85A protein (C and D) or PPD (E and F). Wilcoxon matched-pairs signed rank when compared to baseline (screening visit): ***p < 0005, **p < 005, *p < 05.

Using an ex vivo IFN-γ ELISPOT assay there was a significant increase in the number of Ag85A peptide, Ag85A protein and PPD antigen specific T cells detected 7 days following immunisation with either 1 × 10^7^ (*p* < 0.0005–*p* < 05) or 1 × 10^8^ PFU (*p* < 0.0005–*p* < 05) of MVA85A when compared with baseline (prevaccination) responses (Fig. 3(A)–(F)). Specific T cell frequencies remained detectable and significantly above those measured at baseline for both doses in response to stimulation with 85 A peptides and Ag85A protein at 52 weeks (Fig. 3(A)–(D). In the lower dose group (1 × 10^7^ PFU of MVA85A) PPD specific T cells were not significantly above baseline at 52 weeks but in the higher dose group PPD responses were still significantly higher than at baseline (Fig. 3E and F).

### 3.4 T cell epitope display in response to immunisation with MVA85A

**Fig. 4:**
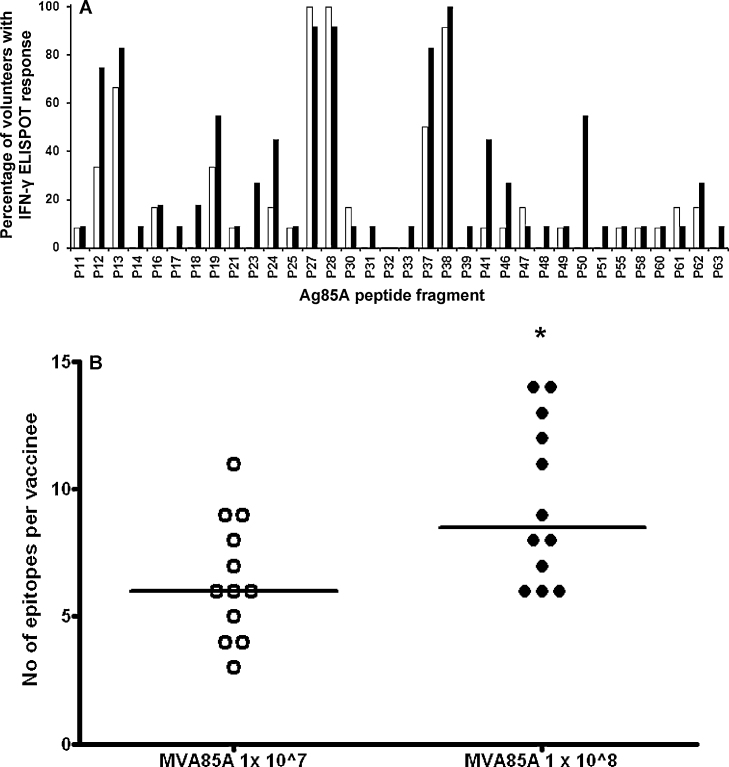
T cell epitope display in adults vaccinated with either 1 × 10^7^ or 1 × 10^8^ PFU of MVA85A. A total of 66, 15-mer peptides overlapping by 10 amino acids (P1–P66) were used to map T cell responses to MVA85A. A) Open bars indicate individual peptide responses to 1 × 10^7^ (*n* = 12) and black bars 1 × 10^8^ PFU (*n* = 12) of MVA85A. B) The overall number of peptides detected in response to MVA85A is significantly higher in volunteers vaccinated with 1 × 10^8^ (black circles) compared to 1 × 10^7^ (white circles) PFU of MVA85A. Responses detected using an ex vivo IFN-γ ELISPOT assay, Mann–Whitney *p < 0.05.

To determine the breadth of epitope response to Ag85A, PBMC collected 7 days following immunisation with MVA85A were stimulated with 66 15mer Ag85A peptides overlapping by 10 amino acids (P1–P66). T cell responses were measured using an ex vivo IFN-γ ELISPOT assay. Immunisation with either 1 × 10^7^ or 1 × 10^8^ PFU of MVA85A induced a broad epitope response with peptides P27 (GKAGC**QTYKWETFLT**), P28 (**QTYKWETFLT**SELPG) and P38 (FVYAGAMSGLLDPSQ) being the most frequently detected epitopes (Fig. 4A). The total number of epitopes detected per volunteer was higher in volunteers receiving 1 × 10^8^ compared to 1 × 10^7^ PFU of MVA85A, (*p* < 0.05; Fig. 4B). Magnetic bead depletion using anti-CD4 and anti CD8 Dynabeads confirmed that all IFN-γ ELISPOT responses were mediated by CD4+ T cells (data not shown).

### 3.5 T cell responses are increased in response to high dose MVA85A when compared to lower doses

**Fig. 5:**
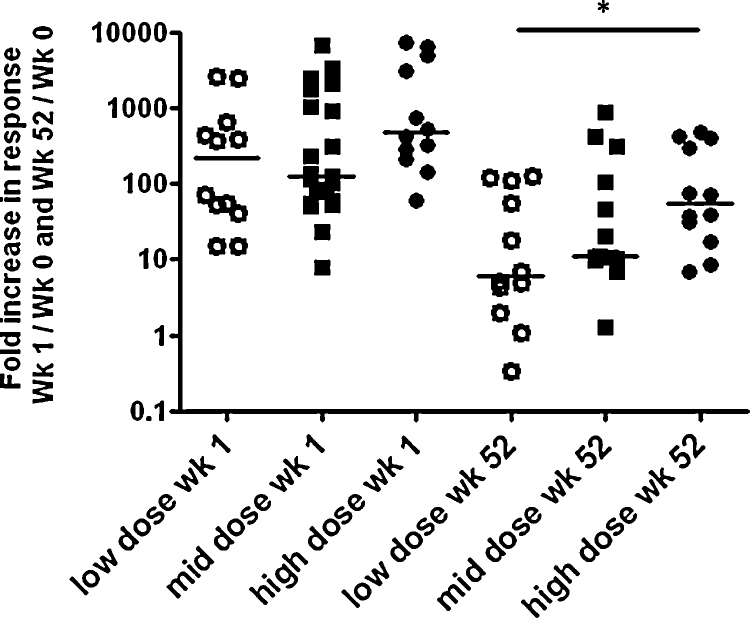
Dose related differences in T cell response to MVA85A. The fold increase in the frequency of antigen specific T cells detected following immunisation with different doses of MVA85A at the peak of response (1 week) and long term (52 weeks) over the screening pre-immunisation responses were calculated for each dose. There was a significant difference in the fold increase between the high and low dose groups at 52 weeks, but no significant difference between high, mid or low doses at 1 week post immunisation.

A comparison was made between the number of antigen specific T cells detected using an IFN-γ ELISPOT assay from volunteers receiving 1 × 10^7^ and 1 × 10^8^ (low and high doses) with previously published data from healthy, previously BCG vaccinated adults receiving 5 × 10^7^ PFU (mid dose) MVA85A [9,10]. High dose MVA85A induced a significantly greater response to Ag85A peptide at 1 week following immunisation when compared to low and mid doses of MVA85A (*p* < 0.002 and *p* < 0.0003; Table 4). At 52 weeks high dose MVA85A induced a greater response than low dose but not mid dose MVA85A (*p* < 0.002; Table 4). The total antigen specific T cell response induced by MVA85A was assessed for each dose by calculating the area under the curve (AUC) from 0 to 24 and 0–52 weeks following immunisation with MVA85A. High dose MVA85A (1 × 10^8^ PFU) induced a significantly greater T cell response than either mid or low dose MVA85A over both 0–24 and 0–52 weeks following immunisation (Table 5). Finally, we calculated the T cell response to MVA85A relative to the screening response. Using this analysis the dose of vaccine given did not have any significant effect on the peak immune response at 1 week following immunisation (Fig. 5). There was however a dose effect at 52 weeks following immunisation with a greater relative response observed in adults receiving the highest dose.

## 4 Discussion

We have previously reported that in BCG-vaccinated UK adults, immunisation with 5 × 10^7^ PFU of MVA85A was well-tolerated and induced a strong T cell response that was maintained until at least 24 weeks following immunisation [10,13]. The optimal vaccine dose, both for safety and immunogenicity, needs to be determined for the further development of MVA85A. Here, we report the results of a dose finding study where we immunised BCG-vaccinated UK adults with either 1 × 10^7^ or 1 × 10^8^ PFU of MVA85A. Both doses were well-tolerated and induced a significant increase in the frequency of Ag85A specific T cells detected at peak (one week) and up to one year following immunisation with MVA85A. When comparing the 2 doses of MVA85A used in this trial with previously published data using an intermediate dose, a clear dose response relationship was observed with a greater frequency of T cells induced both at one and 52 weeks following immunisation in volunteers receiving the higher, 1 × 10^8^ PFU dose. When T cell responses were examined relative to pre-immunisation responses there was no significant effect of dose on the magnitude of response induced at one week following immunisation, however, at one year volunteers who received 1 × 10^8^ PFU of MVA85A had higher numbers of antigen specific T cells detected in peripheral blood. There were no serious vaccine related AEs reported for any volunteer in either the 1 × 10^7^ or 1 × 10^8^ PFU of MVA85A dosing groups. Published studies from other candidate TB vaccines currently in clinical development include Aeras 402 [14], MTB72 [15,16] and H1 [17]. Clinical studies were performed in different populations and IFN-γ was measured using different laboratory assays so direct comparison of the immunogenicity of these vaccine candidates is not possible. Both Aeras 402 and MVA85A have been evaluated using a whole blood ICS assay and in BCG vaccinated adults the median total number of cytokine producing CD4 and CD8 cells in response to Ag85A/B following Aeras 402 was approximately 0.2% of CD4 and 0.3% of CD8 T cells and to the 1 × 10^8^ dose of MVA85A was 0.6% of CD4 and 0.2% of CD8 T cells [14,18]. Using a PBMC ICS assay, both MVA85A and MTB72F induce approximately 800 CD3 + CD4 + CD40L + IFN-γ cells per 10^6^ CD4+ T cells [15,18]. Using a short-term cultured IFN-γ ELISPOT assay which incorporates an overnight expansion of T cells, Van Dissel et al. reported a response of approximately 500 SFU per million sustained to 32 weeks post immunisation [17]. In a direct comparison conducted by four different laboratories the short-term cultured IFN-γ ELISPOT was found to amplify the IFN-γ response 4–10 fold when compared with the 18 h IFN-γ ELISPOT [19]. The IFN-γ response induced by the 1 × 10^8^ dose of MVA85A is therefore higher at weeks 1–4 and at least equivalent at weeks 24 and 52 to the week 32 responses reported for H1 [17,19]. The IFN-γ immune response induced by MVA85A is similar to or greater than that induced by other candidate TB vaccines currently in clinical development, however, IFN-γ alone may not be a correlate of immune protection from disease. MVA85A has now been evaluated in several different populations including those in the UK, Gambia, South Africa and Senegal [4,5,7–10]. Our studies have shown that the AE profile for MVA85A is highly comparable across different populations tested regardless of dose, BCG immunisation status, *MTB* infection status, HIV status, age of participant or country of residence. The frequency of mild or moderate systemic AEs was higher in UK volunteers receiving the 1 × 10^8^ PFU MVA85A dose when compared to the lower doses. Although we have not tested doses higher than 1 × 10^8^ PFU of MVA85A in clinical trials, others have reported an increase in the frequency of severe systemic AEs in adults receiving 5 × 10^8^ PFU of a recombinant MVA construct [16]. An MVA expressing the influenza virus antigens NP and M1 evaluated in UK adults induced severe systemic AEs including nausea/vomiting, malaise or rigours in 5 of 8 volunteers tested [16].

In South African infants a dose finding study with MVA85A found no difference in the magnitude of T cell response induced by 2.5 × 10^7^, 5 × 10^7^ or 1 × 10^8^ PFU of MVA85A up to 6 months following immunisation [4]. In contrast, in UK adults, in the data presented here, we observe a clear dose response relationship with the greatest difference in response observed at 12 months following immunisation. As the magnitude of IFN-γ response induced by even the lowest dose of MVA85A is, at the peak of response at 7 days, near the upper detection limit of the IFN-γ ELISPOT assay it is possible that this assay, at this time point, is not optimal for elucidating differences between doses. We have previously reported a significant effect of MVA85A dose on the induction of IL-17 responses following immunisation with MVA85A in humans [11,20]. IL-17 producing cells were detected at a lower frequency than IFN-γ producing cells and only detected in response to a high dose of 1 × 10^8^ PFU MVA85A. As with IFN-γ, there was no dose-related difference observed in IL-17 responses in infants vaccinated with MVA85A [4]. The lack of dose response in South African infants when compared to UK adults could be due to differences in the maturity of the immune system in adults and infants, differences in environmental exposure or differences in study design as responses were measured up to only 6 months in South African infants, whereas the greatest effect of dose was observed at 12 months in adults.

## Figures and Tables

**Table 1:** Sequences of Ag85A peptides used in the IFN-γ ELISPOT assay.

Sequence-15 mer overlapping by 10	Number	Sequence-15 mer overlapping by 10	Number
MQLVDRVRGAVTGMS	1	VGLSMAASSALTLAI	34
RVRGAVTGMSRRLVV	2	AASSALTLAIYHPQQ	35
VTGMSRRLVVGAVGA	3	LTLAIYHPQQFVYAG	36
RRLVVGAVGAALVSG	4	YHPQQFVYAGAMSGL	37
GAVGAALVSGLVGAV	5	FVYAGAMSGLLDPSQ	38
ALVSGLVGAVGGTAT	6	AMSGLLDPSQAMGPT	39
LVGAVGGTATAGAFS	7	LDPSQAMGPTLIGLA	40
GGTATAGAFSRPGLP	8	AMGPTLIGLAMGDAG	41
AGAFSRPGLPVEYLQ	9	LIGLAMGDAGGYKAS	42
RPGLPVEYLQVPSPS	10	MGDAGGYKASDMWGP	43
VEYLQVPSPSMGRDI	11	GYKASDMWGPKEDPA	44
VPSPSMGRDIKVQFQ	12	DMWGPKEDPAWQRND	45
MGRDIKVQFQSGGAN	13	KEDPAWQRNDPLLNV	46
KVQFQSGGANSPALY	14	WQRNDPLLNVGKLIA	47
SGGANSPALYLLDGL	15	PLLNVGKLIANNTRV	48
SPALYLLDGLRAQDD	16	GKLIANNTRVWVYCG	49
LLDGLRAQDDFSGWD	17	NNTRVWVYCGNGKPS	50
RAQDDFSGWDINTPA	18	WVYCGNGKPSDLGGN	51
FSGWDINTPAFEWYD	19	NGKPSDLGGNNLPAK	52
INTPAFEWYDQSGLS	20	DLGGNNLPAKFLEGF	53
FEWYDQSGLSVVMPV	21	NLPAKFLEGFVRTSN	54
QSGLSVVMPVGGQSS	22	FLEGFVRTSNIKFQD	55
VVMPVGGQSSFYSDW	23	VRTSNIKFQDAYNAG	56
GGQSSFYSDWYQPAC	24	IKFQDAYNAGGGHNG	57
FYSDWYQPACGKAGC	25	AYNAGGGHNGVFDFP	58
YQPACGKAGCQTYKW	26	GGHNGVFDFPDSGTH	59
GKAGCQTYKWETFLT	27	VFDFPDSGTHSWEYW	60
QTYKWETFLTSELPG	28	DSGTHSWEYWGAQLN	61
ETFLTSELPGWLQAN	29	SWEYWGAQLNAMKPD	62
SELPGWLQANRHVKP	30	GAQLNAMKPDLQRAL	63
WLQANRHVKPTGSAV	31	AMKPDLQRALGATPN	64
RHVKPTGSAVVGLSM	32	LQRALGATPNAVPAP	65
TGSAVVGLSMAASSA	33	GATPNAVPAPQGA	66

**Table 2:** Demograpahic characteristics of UK adults vaccinated with MVA85A.

	MVA85A dose		
	1 × 10^8^ PFU *n* = 12	1 × 10^7^ PFU *n* = 12	5 × 10^7^ PFU *n* = 17–31
Male, no (%)	4 (33)	4 (33)	12 (39)
Median age, years (range)	25 (19–32)	27 (21–42)	27 (21–54)
Continent of birth, no (%)			
UK	5 (42)	8 (67)	27 (87)
Africa	3 (25)	3 (25)	1 (3)
Other	4 (33)	1 (8)	3 (10)
Healthcare worker, no (%)	6 (50)	7 (67)	5 (16)
Interval between BCG and MVA85A, no			
0–5 years	3	0	5
6–10 years	2	5	3
11–15 years	3	2	0
16–20 years	0	0	0
>20 years	4	5	9

**Table 3:** Adverse events reported following immunisation with different doses of MVA85A.

	Vaccine dose		
	1 × 10^8^ PFU Subjects, *n* (%)	1 × 10^7^ PFU Subjects, *n* (%)	5 × 10^7^ PFU Subjects, *n* (%)
**Local AE**			
Erythema	12 (100%)	12 (100)	31 (100%)
Pain	12 (100%)	6 (50%)	27 (87%)
Pruritus	8 (67%)	6 (50%)	18 (58%)
Scaling	3 (25%)	6 (50%)	29 (94%)
Swelling	12 (100%)	12 (100%)	31 (100%)

**Systemic AE**			
Arthralgia	7 (58%)	1 (8%)	6 (19%)
Documented fever	5 (42%)	0	3 (10%)
Felt feverish	9 (75%)	2 (17%)	8 (26%)
Headache	10 (83%)	1 (8%)	11 (35%)
Lymphadenopathy	2 (17%)	0	4 (13%)
Malaise	7 (58%)	2 (17%)	12 (39%)
Myalgia	9 (75%)	0	11 (35%)
Nausea or vomiting	4 (33%)	1 (8%)	5 (16%)
Diarrhoea	1 (8%)	0	2 (6%)
Vasovagal syncope	0	0	1 (3%)

**Table 4:** Peak and long term T cell responses induced by 1 × 10^7^ and 1 × 10^8^ PFU of MVA85A (low and high dose) in comparison to the previously published dose of 5 × 10^7^ PFU of MVA85A (mid dose). Values in the table are spot-forming units (SFU) per million PBMC.

	Group 1 (low dose)	Group 2 (mid dose)	Group 3 (high dose)	*p* value*	Difference in medians (95% CI^a^) [*p*-value**]		
	Median (IQR) *n* = 12	Median (IQR) *n* = 21	Median (IQR) *n* = 12		Groups 1 vs. 2	Groups 2 vs. 3	Groups 1 vs. 3
**Week 1 (peak response)**							
85A peptides	2532 (573–3659)	2147 (1237–4982)	6493 (4896–7152)	0.0006	−519 (−2027, 963) [0.55]	−3230 (−4750, −1658) [0.002]	−3939 (−4995, −2314) [0.0003]
Ag85A	957 (294–1657)	790 (438–1653)	1653 (1630–1657)	0.10	–	–	–

	Group 1 (low dose)	Group 2 (mid dose)	Group 3 (high dose)	*p* value*	Difference in medians (95% CI^a^) [*p*-value**]		
	Median (IQR) *n* = 12	Median (IQR) *n* = 13	Median (IQR) *n* = 12		Groups 1 vs. 2	Groups 2 vs. 3	Groups 1 vs. 3
**Week 52 (long term response)**							
85A peptides	115 (66–278)	506 (227–881)	413 (388–772)	0.0017	−343 (−757, −103) [0.002]	−37 (−277, 260) [0.91]	−337 (−535, −213) [0.002]
Ag85A	85 (54–162)	237 (170–503)	337 (242–552)	0.0006	−169 (−420, −67) [0.002]	−52 (−193, 173) [0.50]	−240 (−460, −123) [0.0005]

IQR: inter quartile range.

a Estimated using the Binomial method.

* Kruskall–Wallis test for difference between the 3 groups.

** Mann–Whitney test for pairwise comparisons.

**Table 5:** Total antigen specific T cell responses induced by 1 × 10^7^ and 1 × 10^8^ PFU of MVA85A (low and high dose) in comparison to the previously published dose of 5 × 10^7^ PFU of MVA85A (mid dose). Values in the table are spot-forming units (SFU) per million PBMC.

	Group 1 (Low dose)	Group 2 (Mid dose)	Group 3 (High dose)	*p* value*	Difference in medians (95% CI^a^) [*p*-value**]		
	Median (IQR) *n* = 12	Median (IQR) *n* = 21	Median (IQR) *n* = 12		Groups 1 vs. 2	Groups 2 vs. 3	Groups 1 vs. 3
**Area under the curve weeks 0–24**							
85A peptides	15,444 (6746, 20,107)	16,317 (9363, 32,646)	41,575 (27,551, 52,012)	0.002	−3953 (−14,596, 3944) [0.51]	−19,273 (−33,253, −7887) [0.006]	−26,200 (−39,270, −11,994) [0.0003]
Ag85A	7964 (3967, 9958)	5055 (3258, 9372)	19,172 (15,119, 23,261)	0.0004	964 (−3019, 4270) [0.56]	−13,083 (−17,189, −5877) [0.0004]	−12,410 (−17,128, −7177) [0.0007]

	Group 1 (Low dose)	Group 2 (Mid dose)	Group 3 (High dose)	*p* value*	Difference in medians (95% CI^a^) [*p*-value**]		
	Median (IQR) *n* = 12	Median (IQR) *n* = 13	Median (IQR) *n* = 12		Groups 1 vs. 2	Groups 2 vs. 3	Groups 1 vs. 3
**Area under the curve weeks 0–52**							
85A peptides	18,166 (9238, 30,488)	31,760 (16,736, 67,808)	60,797 (44,736, 77,275)	0.001	−10,275 (−42,266, 1396) [0.11]	−26,918 (−45,813, 139) [0.06]	−40,226 (−59,224, −23,862) [0.0002]
Ag85A	9714 (6756, 15,874)	12,937 (8310, 19,942)	31,424 (25,079, 41,581)	0.001	−4292 (−12,551, 1961) [0.25]	−16,493 (−25,916, −4274) [0.01]	−22,083 (−30,504, −13,383) [0.0003]

IQR: inter quartile range.

a Estimated using the Binomial method.

* Kruskall–Wallis test for difference between the 3 groups.

** Mann–Whitney test for pairwise comparisons.
